# Comparative Study of ZnO-and-TiO_2_-Nanoparticles-Functionalized Polyvinyl Alcohol/Chitosan Bionanocomposites for Multifunctional Biomedical Applications

**DOI:** 10.3390/polym15163477

**Published:** 2023-08-19

**Authors:** Annu, Zafar Iqbal Bhat, Khalid Imtiyaz, M. Moshahid A. Rizvi, Saiqa Ikram, Dong Kil Shin

**Affiliations:** 1 Thin-Film Engineering and Materials Laboratory, School of Mechanical Engineering, Yeungnam University, Gyeongsan 38541, Republic of Korea; 2 Bio/Polymers Research Laboratory, Department of Chemistry, Jamia Millia Islamia, New Delhi 110025, India; 3 Department of Biosciences, Jamia Millia Islamia, New Delhi 110025, India

**Keywords:** bionanocomposites, zinc oxide NPs, titanium dioxide NPs, antioxidant, skin cancer A431, polyvinyl alcohol, chitosan, biomedical

## Abstract

This study aimed to synthesize chitosan/polyvinyl alcohol (CS/PVA)-based zinc oxide (ZnO) and titanium dioxide (TiO_2_) hybrid bionanocomposites (BNCs) and observe their comparative accomplishment against the skin cancer cell line, A431, and antioxidant potential. CS was blended with PVA to form polymeric films reinforced with the immobilization of ZnO and TiO_2_ nanoparticles (NPs), separately. The optimization of the BNCs was done via physicochemical studies, viz. moisture content, swelling ratio, and contact angle measurements. The free radical scavenging activity was observed for 1,1-diphenyl-2-picryl-hydrazyl, and the antibacterial assay against the *Escherichia coli* strain showed a higher zone of inhibition. Furthermore, the anticancer activity of the synthesized BNCs was revealed against the skin cancer cell line A431 under varying concentrations of 50, 100, 150, 200, and 300 μg/mL. The anticancer study revealed a high percent of cancerous cell inhibition (70%) in ZnO BNCs as compared to (61%) TiO_2_ BNCs in a dose-dependent manner.

## 1. Introduction

With the recent advancement of nanotechnology and its applications in biomedical fields, nanoengineering has become one of the most vibrant achievements in the field of science in the current century. Nanotechnology not only advanced the targeted drug delivery mechanism, drug manufacturing, and diagnostics application, but also made it conceivable for the simultaneous detection and cure of a wide range of life-threatening diseases like cancer [1,2]. Presently, cancer is the second most life-threatening disease following cardiac diseases, accounting for the death of around more than 10 million people around the world as per a World Health Organization report [3]. Among cancers, skin cancer is recorded as the seventeenth most common cancer worldwide, leading among Australians. The incidences of both 2–3 million (non-melanoma) and 1.3 lacs (melanoma) type skin cancers increase globally each year. In 2020, approximately 325,000 new cases were diagnosed and 57,000 people died from (melanoma) skin cancer [3,4].

Although cancer treatments are available as chemotherapy or surgical interventions, these procedures show some serious adverse effects [4]. Therefore, thrust areas of research in this field involve novelty in procedures or anticancer agents with high effectiveness, low toxicity, excellent biocompatibility, and biodegradability. Therefore, subject to all these concerns, nanotechnology emerges as an important field growing rapidly with novel advancements and showing significant improvement in drug delivery, bioavailability, imaging, and chemotherapy, with minimization of the adverse effects [5,6]. Different BNCs, including various nanosized materials such as graphene oxide, titanium oxide, zinc oxide, gold, silver, nanofillers, etc., were explored for the cancer treatment [7,8,9].

Among different nanosized materials, zinc oxide (ZnO) and titanium dioxide (TiO_2_) induce potent anticancer activities even in very minute concentrations [10]. Further, the combination of these nanomaterials with polymeric materials enhances their activities while minimizing their adverse effects. Chitosan (CS), a natural polysaccharide having inherent biomedical properties, has been widely used for biocomposite formation [11,12,13] With β-1, 4-glycosidic linkage, CS exhibits excellent properties such as non-toxicity, biodegradability, biocompatibility, mucoadhesion, chelating ability, and excellent film-forming capacity and is hence utilized in textile, wastewater treatment, medical domains, etc. [14]. Moreover, CS films are also known for their selective permeability to CO_2_ and O_2_ gases; however, CS films are a poor barrier to moisture, owing to their hydrophilic nature. Therefore, blending with a biodegradable synthetic polymer can be used to improve the water resistance, mechanical strength, and thermal stability of CS films [15]. PVA possesses outstanding properties such as chemical stability, non-toxicity, high hydrophilicity, high mechanical strength, film-forming ability, and most importantly, biodegradability and biocompatibility [16]. Hence, the blend membranes of CS and PVA were put forth as a good combination with enhanced intrinsic properties of the films [17,18,19].

Chitosan nanosilver hybrid BNCs with enhanced antimicrobial, antioxidant, and anticancer activities have been synthesized by Annu et al. through a green and sustainable route. Chitosan/polyvinyl alcohol/ZnO BNCs films have already been reported [20,21] for biocidal activities and the nanofibrous mats have been developed for diabetic wound healing [22], and their in vivo studies showed accelerated wound healing. Chitosan-TiO_2_ composite materials and their cytotoxic effects have been reported [23,24,25] against different cell lines.

**Figure 1:**
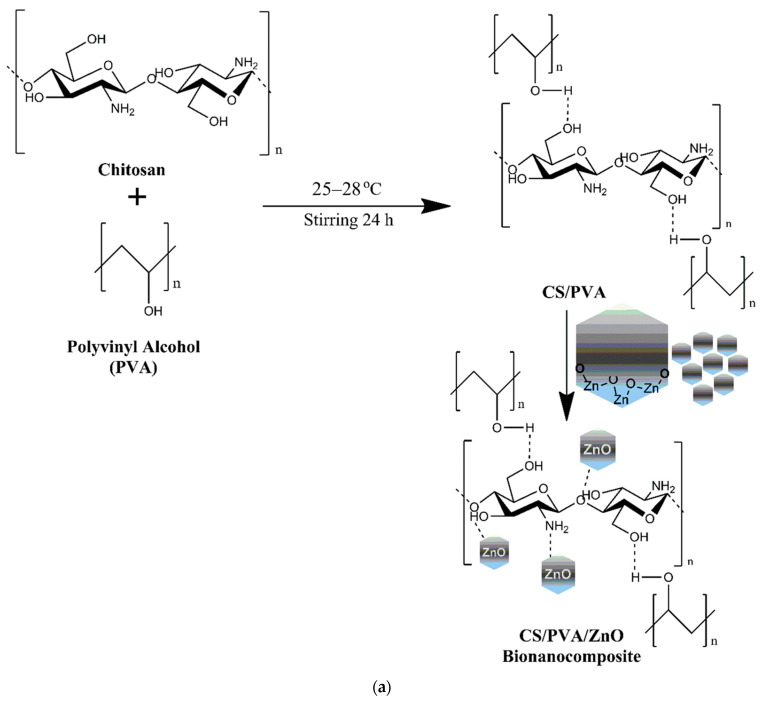

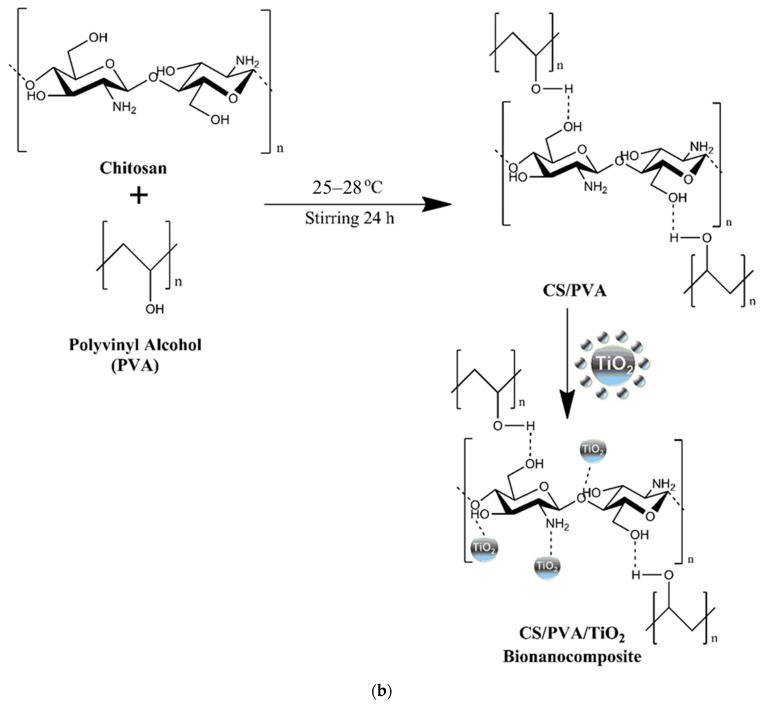
Probable interaction of (**a**) ZnO NPs and (**b**) TiO_2_ NPs with CS/PVA blended polymers.

In this study, ZnO and TiO_2_ NPs have been incorporated in the CS/PVA polymeric blended films to fabricate their respective CS/PVA/ZnO (CPZ) and CS/PVA/TiO_2_ (CPT) BNCs and to investigate their biomedical efficacy in terms of their scavenging activity, antibacterial activity, as well as anticancer activity against the skin cancer cell line A431. This is among the first comparative study where ZnO and TiO_2_ NPs were studied together for their skin cancer activity. The schematic representation and probable interaction of both the NPs are depicted in Figure 1a,b, respectively. The nanoparticles interact with both –OH and –NH_2_ groups via electrostatic interaction. This interaction can be explained by the hard–soft acid–base (HSAB) principle, where a hard acid has a stronger affinity for a hard base. Thus, a metal ion in a higher oxidation state (usually considered as a hard acid, Zn^2+^, and Ti^4+^) can interact strongly with a hard base (usually a small and non-polarizable atom-like O and N). Further, the interaction becomes clear from the FTIR spectral analysis, where a clear shifting was observed in the absorption intensities of both -OH and -NH_2_ functional groups.

## 2. Experimental

### 2.1. Materials

Chitosan (MW = 164.05 kDa; >75% degree of deacetylation) from Sigma-Aldrich Chemicals Pvt. Ltd., Bangalore, India; PVA (MW = 125,000 g/mol approx.) from SD Fine-Chem limited (SDFL), Mumbai, India; Zinc Oxide nanopowder (ZnO, 99.5% purity, 10–80 nm size); Titanium dioxide nanopowder (TiO_2_, 99.9% purity, 10–25 nm size) was purchased from NANOSHEL, India; and Glacial acetic acid of analytical grade (99–100%) was purchased from Fischer Scientific, Mumbai, India. Chemicals were used as purchased and without further purification. Glassware was thoroughly washed with chromic acid followed by double distilled water (DDW) and dried in the oven for further use.

### 2.2. Synthesis of CS/PVA Blend Solution

The 1% CS solution (*w*/*v*) was prepared by dissolving CS in a 1% (*v*/*v*) acetic acid solution in a 100 mL Erlenmeyer flask by stirring for 2 h. The 5% PVA (*w*/*v*) solution was prepared using DDW in a 100 mL Erlenmeyer flask by stirring for 1 h. Both solutions were mixed slowly with continuous stirring on a magnetic stirrer at room temperature.

### 2.3. Fabrication of CPZ and CPT BNCs

The ZnO and TiO_2_ nanopowders were individually dissolved in DDW to prepare the 0.1 and 0.5% *w/v* solutions, separately. The NPs solution was poured slowly into the blended solution of CS/PVA with continuous stirring on a magnetic stirrer. All the blended solutions, containing NPs, were poured into the glass Petri dishes for casting and then kept in an oven at 40 °C to obtain the desired CPZ and CPT BNC films as CPZ1 (0.1%) and CPZ2 (0.5%), and CPT1 (0.1%) and CPT2 (0.5%), respectively, as shown in Table 1. The films of CS, PVA, and CS/PVA were also cast in the same manner.

## 3. Characterization of the BNCs Thin Films

The synthesized BNCs were characterized using standard methods and techniques, such as flexibility, moisture content analysis, swelling, water contact angle (WCA), UV-visible spectroscopy, FT-IR, XRD, HR-FESEM, and TEM to assess their physicochemical characteristic features. This section describes the sample preparation, methodologies, and assessment of the synthesized BNCs.

### 3.1. Physical Measurements of CPZ and CPT BNCs

#### 3.1.1. Physical Analysis of the BNCs

##### Appearance, Thickness, and Flexibility

The thickness of CS, PVA, CS/PVA, CPZ, and CPT BNC films was measured by using a handheld digital micrometer Vernier caliper (aerospace) with an accuracy of ±0.01 mm. The Vernier caliper was calibrated before use. The thickness of the films was measured at six random locations of the film. The measurements were repeated thrice, and the average thickness is reported.

##### Moisture Content

The moisture content of ZnO and TiO_2_ BNC films of different compositions was determined following the procedure mentioned in [26]. Briefly, the weight loss of the films was measured after drying them in an air-circulating oven at 100 ± 5 °C for 24 h. All the samples were analyzed in triplicate with accurate weighing, before and after drying the films.

The moisture content was calculated as the percentage of water in the films using Equation (1):

(1) $$Moisture\;content\;\%=\frac{\left[\left(W_{w}-W_{d}\right)\right]}{W_{w}}\times 100$$

where *W_w_* is the initial weight of films at room temperature and *W_d_* is the weight of films after drying.

##### Swelling Behavior

All the CS/PVA-based ZnO and TiO_2_ BNC films were examined for their swelling behavior by immersing the film samples in a buffer solution of pH 6.8 at 25 °C. The swollen film samples were weighed every 5 min up to equilibrium, and then immediately after the excessive surface water was extracted from the film samples by gently tapping the surface on a filter paper [27]. Each sample was analyzed thrice, and the average value was considered to be the swelling ratio, which can be calculated according to Equation (2):

(2) $$Swelling\;ratio\;\left(\%\right)=\frac{\left[(W_{s}-W_{d})\right]}{W_{d}}\times 100\;$$

where *W_s_* and *W_d_* are the weights of the swollen and dry film samples.

##### Hydrophilicity

The surface properties of CS/PVA-based CPZ and CPT BNC films were also examined for hydrophilicity by employing static contact angle measurements using the optical contact angle analyzer, SEO Phoenix 150, at room temperature as in our previous study [28]. The film samples were cut into coupons of dimensions 10 × 10 mm. The coupons were kept on the slide surface and a DIW (5 µL) drop was put onto the surface of the film using a micropipette to measure the water contact angle of the samples.

#### 3.1.2. Functional Analysis of the BNCs

##### UV-Visible Spectroscopy

The CS/PVA-based ZnO and TiO_2_ BNC films were characterized for maximum absorption intensity in the range of 200–700 nm under UV-vis spectroscopy by using a Shimadzu 1800 spectrophotometer (Tokyo, Japan). DIW was taken as a reference.

##### FT-IR Spectroscopy

The FT-IR analysis of the samples was carried out to determine the interaction of CS with PVA and ZnO and TiO_2_ NPs with the CS/PVA blend. The FT-IR spectra of the BNCs films were recorded by using a Bruker Tensor 37 spectrometer (Billerica, MA, USA) in the range of 600–4000 cm^−1^.

##### X-ray Diffraction Studies

The wide-angle X-ray diffraction was observed to detect the crystalline phase of CS/PVA-based ZnO and TiO_2_ films with the help of a Rigaku Ultima IV X-ray Diffractometer (Tokyo, Japan) furnished with a graphite monochromator and Cu Kα radiation (λ = 1.5415 Å), functioning at 30 mA, 45 kV, and 25 °C. The films were mounted on a sample holder and scanned at a rate of 8°/min in the 2θ range (5°–80°).

#### 3.1.3. Morphological Analysis of the BNCs

##### HR-FESEM Analysis

HR-FESEM analysis of ZnO- and TiO_2_-immobilized CS/PVA films were carried out using the ZEISS, Model V5.05 (Sigma, St. Louis, MO, USA). The thin film samples were pasted over carbon tape and sputter coated with gold to analyze their morphology using HR-FESEM.

##### TEM Analysis

The TEM analysis of the BNCs was performed on an electron microscope, model number JEOL JEM-1400 (Tokyo, Japan), at an accelerating voltage of 120 kV. Specimens for the TEM measurements were prepared by depositing a drop of a colloid solution of BNCs on a 400-mesh copper grid coated by an amorphous carbon film and evaporating the solvent by keeping the sample grid inside the oven at a temperature of 30 °C.

### 3.2. Statistical Analysis of the Results

All the experiments were carried out in triplicates. The results are reported as the mean, and the standard deviation of means. The one-way analysis of variance (ANOVA) was performed. Significant differences between the different samples were analyzed using the Tukey test. A *p*-value less than 0.05 was considered statistically significant.

### 3.3. Biological Assays

The biological activity of the synthesized BNCs was considered in terms of antibacterial, antioxidant, and anticancer activities. Further, the DPPH and MTT assays were carried out to confirm the antioxidant and anticancer activities of the BNCs, respectively. This section states the procedure and methodologies carried out to perform the biological assays of the BNCs.

### 3.4. Antibacterial Studies

The antibacterial activity of CPZ and CPT BNCs was evaluated by using the disc diffusion method. Briefly, a culture of bacteria, *Escherichia coli*, was prepared by growing a single colony overnight in a nutrient broth and adjusting the turbidity to the 0.5 McFarland standard. The bacterial test pathogens (100 µL) were spread onto 25 mL Mueller–Hinton agar plates. Thereafter, 50 µL of different concentrations (50, 100, 150, 200, and 300 μg/mL) of the BNCs solution and 50 μL of antibiotics (Ampicillin) of the 10 μg/mL concentration were poured on the diffusion discs. The inhibitory effects of BNCs were evaluated by measuring the respective zones of inhibition around each disc containing a test compound or standard. All the experiments were simultaneously performed in triplicates.

### 3.5. Antioxidant Studies

#### DPPH Assay

The 1,1-diphenyl-2-picryl-hydrazyl (DPPH) assay was performed using the methods described by Braca et al. [29]. Briefly, various concentrations (1 mL) of the sample/standard (100–1000 μg/mL) were added to 2 mL of the 0.004% methanol solution of DPPH, and the mixtures were vortexed vigorously. The control was prepared as mentioned above except for the analyte/standard. Methanol was used for background subtraction. The samples were then incubated at room temperature for 30 min in the dark, and the absorbance was recorded at 517 nm. The lower absorbance of the reaction mixture indicates higher free radical scavenging activity [30]. Ascorbic acid and butylated hydroxyanisole (BHA) were taken as known free radical scavengers. The percentage inhibition activity was calculated by using Equation (3).

(3) $$\%\;Scavenging=\frac{AC-AS}{AC}\times 100$$

where *AC* was the absorbance of the control and *AS* was the absorbance in the presence of the sample or the standard.

### 3.6. Anticancer Studies

Cell inhibition testing was done using MTT on A431 (the human skin cancer cell line). The cancer cell lines were procured from NCCS Pune, India. The cell lines were cultured in Dulbecco’s minimum essential medium (DMEM) with 10% fetal bovine serum (FBS) (Gibco, Invitrogen, Grand Island, NY, USA), 10 µg/mL of penicillin, and 10 µg/mL of streptomycin. The medium was changed every three days. The cells were harvested using 0.25% trypsin and 0.1% EDTA and seeded onto 96-well plates at 5000 cells/well. Briefly, 10,000 cells/well were seeded into flat-bottom 96-well plates (150 µL/well) in triplicates, allowing them to attach and grow. The cells were incubated for 24 h and subsequently treated with varying concentrations of the compounds ranging from 10 µM to 80 µM. After 48 h of treatment, the medium was removed and the cells were incubated with 20 μL of MTT (5 mg/mL in a phosphate buffer saline (PBS)) in a fresh medium for 4 h at 37 °C. Formazan crystals formed via the mitochondrial reduction in MTT were solubilized in DMSO (150 µL/well) and quantification was performed by reading the absorbance at 540 nm after an incubation period of 15 min on the iMark Microplate Reader (Bio-Rad, Hercules, CA, USA).

## 4. Results and Discussion

### 4.1. Physical Measurements of BNC Films

#### Appearance and Thickness

Visually, all the films were homogenous, uniform without any bubbles or brittle areas, and were peeled out easily from the Petri dishes. The visual representations of all the films are shown in (Supplementary Figure S1). The film thickness is an important criterion for determining the physical properties of the films. The CS linear chain possesses hydration layers having water molecules that prevent the chain approximation, and, hence, yield thick films. Table 2 reveals that the pure PVA film was the thickest (0.16 mm ± 0.01) film, and CPT1 and CPT2 were delicate and the thinnest as compared to CPZ1, CPZ2, and the other biopolymeric films.

### 4.2. Moisture Content

**Figure 2:**
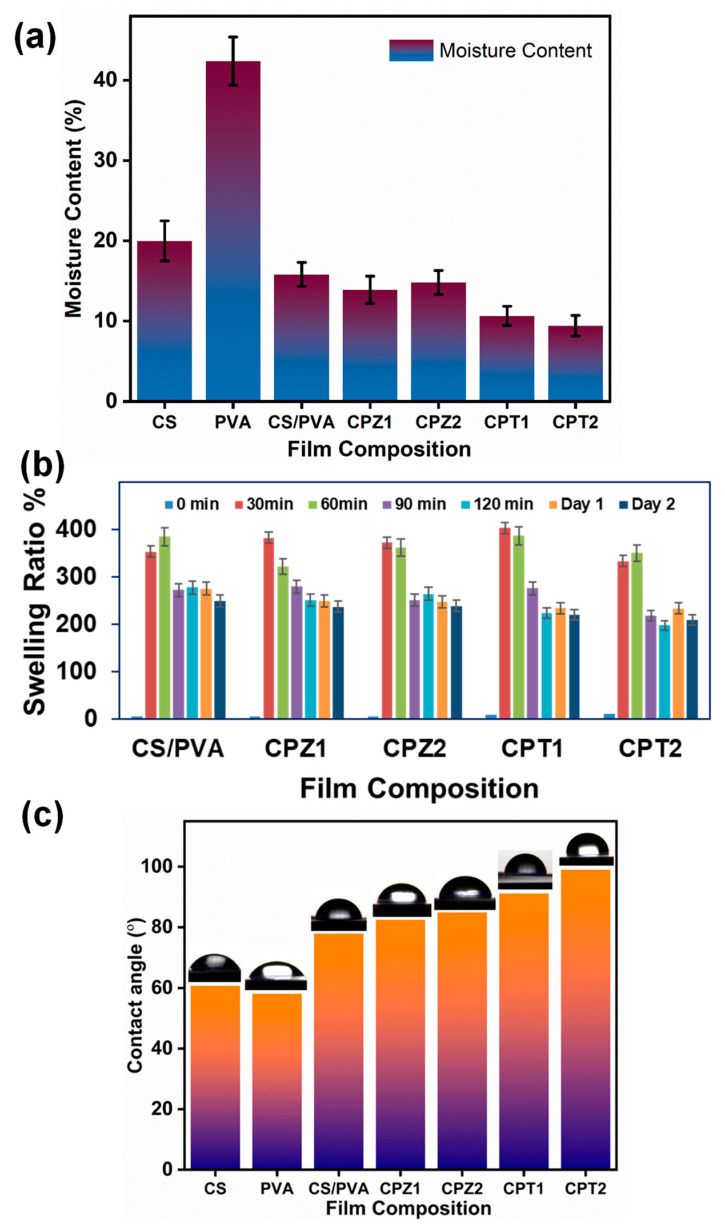
(**a**) Moisture content %, (**b**) swelling ratio %, and (**c**) contact angle measurements of CS-based BNCs. Results are shown as the mean ± SD (*n* = 3), different letters in the superscript show significant differences at *p* < 0.05.

The moisture content is an important trait for the characterization of the thin films developed especially for anticancer applications. The films must sustain an appropriate behavior to restrict moisture. This will help to protect the cargo from moisture. The moisture content study was conducted for CS/PVA blended film and BNC films (Figure 2a). The pure CS films and pure PVA are shown to possess more moisture content percentage, indicating their hydrophilic nature with the highest (42.4 ± 1.73%, results are shown as the mean ± SD (*n* = 3)) in pure PVA as compared to the other composite films. CS/PVA blended films exhibited 15.8 ± 2.2% moisture content, which shows the intermediate moisture content percent of pure CS and PVA and hence affirms their interaction. Aside from that, CPZ1 and CPZ2 BNCs were found to be 13.9 ± 0.23% and 14.8 ± 0.36%, respectively, compared to CS/PVA, whereas CPT1 and CPT2 exhibited the least percentage of 10.6 ± 0.50% and 9.4 ± 0.32%, respectively, of moisture content. This is due to the immobilization of ZnO and TiO_2_ metal NPs in the polymeric matrix, which makes it less capable of retaining moisture in the BNC films.

### 4.3. Swelling Behavior

Swelling studies were examined for developed composite films at a medically suitable pH of 6.8 as shown in Figure 2b. The CS is polycationic and hydrophilic in an acidic medium and allows good miscibility of CS and PVA to get CS/PVA blended films. However, owing to high hydrophilicity, the pure PVA films were completely dissolved in the solution before 30 min of observation were reached. On the contrary, the swelling ratio of the BNC films initially increased up to 1 h and then decreased on day 2. CPZ1 and CPZ2 were shown to have more swelling as compared to the TiO_2_ BNC films, which may be due to the difference in structure and the availability of free –OH groups for affinity towards water molecules. In general, the swelling ratio was greatly influenced by NPs in the BNC films, which was probably due to the interaction of the –NH_2_ and –OH groups of CS/PVA with rigid metal structures via electrostatic interaction, thereby reducing their water uptake capacity till day 2. It was already reported that the swelling degree of polymeric films with the incorporation of metal NPs shows a reduced water uptake due to the formation of a three-dimensional metal oxide network in the polymeric matrix [31].

### 4.4. Hydrophilicity

The hydrophilicity of a material is a trait that approves its affinity for water. The WCA is a definite way to verify the hydrophilicity of a material. The hydrophilicity is higher in case the WCA is lower, and a lower hydrophilicity for materials shows a higher WCA. The low WCA indicates good wettability, reflecting its hydrophilic property as observed for pure PVA (58.09°) and slightly less for pure CS (60.67°), as shown in Figure 2c. This is mainly due to the presence of hydroxyl groups in PVA which undergo hydrogen bonding with the water molecules, and hence, have higher hydrophilic properties than CS. The CS/PVA blended film was observed to be hydrophobic as compared to their pristine films due to the balanced –NH_2_ and –OH groups; meanwhile, CS behaves hydrophobically with PVA, thereby increasing the contact angle to 76.49°. As it has already been reported that CS films blended with metal or other material can alter the wettability of the films, for instance, CS with silver NPs exhibited a high contact angle attributed to the hydrophobicity of the film [32]. However, it has been found that the metal oxide BNCs, CPZ1 (82.46), and CPZ2 (84.88) possess a higher WCA as compared to CS/PVA films, but less than that of CPT1 (91.15°) and CPT2 (99°) BNCs, which is attributed to low wettability than the latter one, indicating their hydrophobic property with the polymeric material. This may be due to the cross-linking of ZnO and TiO_2_ NPs within the blended polymer matrix, which leads to the reduction in the availability of the –OH group for corresponding hydrogen bonding in the respective BNCs films, which become hydrophobic [31].

### 4.5. UV-Visible Spectroscopy

**Figure 3:**
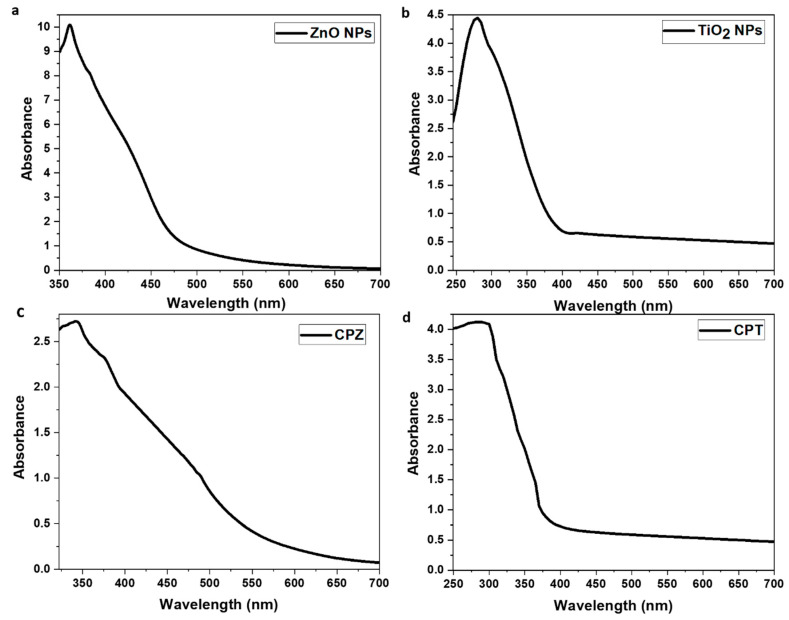
UV spectra of (**a**) ZnO NPs, (**b**) TiO_2_ NPs, (**c**) CPZ, and (**d**) CPT BNC.

The UV-visible absorption spectra of metal oxide BNCs are depicted in Figure 3. As evident from Figure 3a,b, both the metal oxide (ZnO and TiO_2_) NPs show their absorption spectra at 362 nm and 285 nm, respectively. However, the absorption of pure CS was found at a lower wavelength (below 250 nm) and a low intensity without any obvious peak due to the absence of a double bond conjugated system (Supplementary Figure S2). In the case of CPZ BNC, the absorption peak was shifted to the lower wavelength at 342 nm with a broad shoulder as compared to microcrystalline ZnO (362 nm), indicating a blue-shift in wavelength which revealed the reduced size of NPs has been impregnated inside the films (Figure 3c). Moreover, the variation in intensity indicates the varied number of NPs present in the sample. The literature has reported the size-dependent behavior of ZnO NPs with increased and decreased wavelengths [33]. Meanwhile, TiO_2_ NPs show absorbance at around 285 nm, whereas, in the case of CPT BNC, a broad absorption peak was observed at a longer wavelength, indicating a red-shift in wavelengths with less intensity than the former and shows the impregnation of TiO_2_ NPs inside the polymeric matrix (Figure 3d). Saravanan et al. revealed a similar absorption edge of the TiO_2_ nanocomposite in the visible region [34].

### 4.6. FT-IR Spectroscopy

**Figure 4:**
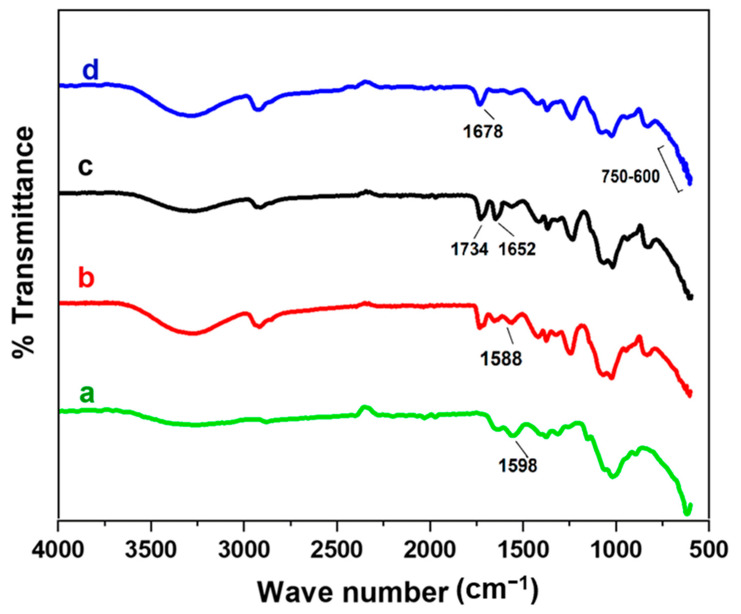
FT-IR spectra of (a) CS, (b) CS/PVA, (c) CPT, and (d) CPZ BNCs.

Figure 4 shows the FT-IR spectra of CS, CS/PVA, and metal oxide BNCs. As depicted in Figure 4a, CS shows a broad peak of the –OH and –NH_2_ stretching vibration from 3480 to 3250 cm^−1^. The peak at 2922 cm^−1^ and 2862 cm^−1^ was due to the presence of alkyl –C-H and –N-H groups; the peaks at 1648 cm^−1^ and 1598 cm^−1^ confirm the –C-O stretching and –N-H bending vibrations of amide I and amide II, respectively; the peak at 1377 cm^−1^ represents the –C-H bending vibrations; the peak at 1322 cm^−1^ was due to the presence of –the C-N stretching vibration of amide III; the peak at 1066 cm^−1^ shows a –C-O-C stretching vibration of the glycosidic linkage; and the peak around 618 cm^−1^ indicates the crystallinity in CS [19]. The FT-IR spectrum of CS/PVA blended films shows broadband at 3450–3065 cm^−1^, which is less in the wave numbers attributed to the –OH group in the blended film [19]. Aside from that, the alkyl group at 2925 cm^−1^ and peaks at 1323 cm^−1^ and 1085 cm^−1^ were observed due to the –O-H in-plane vibrations and –C-O out-of-plane bonding, respectively, along with shifting in the –N-H bending vibration, from 1598 cm^−1^ of amide II to 1588 cm^−1^ in the CS/PVA blended film illuminating the interaction between the –O-H and –N-H groups of CS with the –O-H group of PVA.

The FT-IR spectra of both CPT and CPZ BNCs have shown all the characteristic peaks of CS/PVA, which significantly shifted towards lower frequencies attributed to the interaction among CS/PVA and metal oxide NPs via hydrogen bonding. In the case of CPT BNC, the broad peak of the –O-H stretching vibration was observed at 3500–3050 cm^−1^ which is attributed to the involvement of the –OH group of CS/PVA in the BNC. Further, the first characteristic peak of Ti-O was observed at 1734 cm^−1^, showing the involvement of the –OCNH_2_ group in the electrostatic interaction of PVA with Ti-O and CS as well. The second characteristic peak at 1652 cm^−1^, along with shifting in the wavenumber of CS/PVA at 1372, 1320, and 1081 cm^−1^, was also observed to confirm the incorporated TiO_2_ NPs into the blended polymeric matrix. Additionally, the peaks at 636, 629, and 609 cm^−1^ correspond to the Ti–O stretching vibration, and the –OH group of CS/PVA was also observed to bring intermolecular hydrogen bonding interactions [35]. This implies the immobilization of TiO_2_ NPs into the CS/PVA blended polymeric matrix. Similarly, in the case of CPZ BNCs, the broad peak in the range of 3500–3000 cm^−1^, which attributed to the overlapping of the –O-H and –N-H stretching vibration, which demonstrated the intermolecular and intramolecular –OH group and hydrogen bonding with CS/PVA. The peak observed at 1678 cm^−1^ corresponds to the –C-O stretching vibration on the surface of ZnO NPs due to the interaction with CS/PVA. Also, similar peaks were observed at 1374 cm^−1^ and 1077 cm^−1^, which attributed to the –COO and –C-O-C- stretching vibrations, respectively. This indicates the interactions between the glycosidic linkage of CS/PVA. The overlapped peaks in the 750–500 cm^−1^ range were attributed to the Zn-O stretching vibration as reported in the literature [36].

### 4.7. XRD Studies

**Figure 5:**
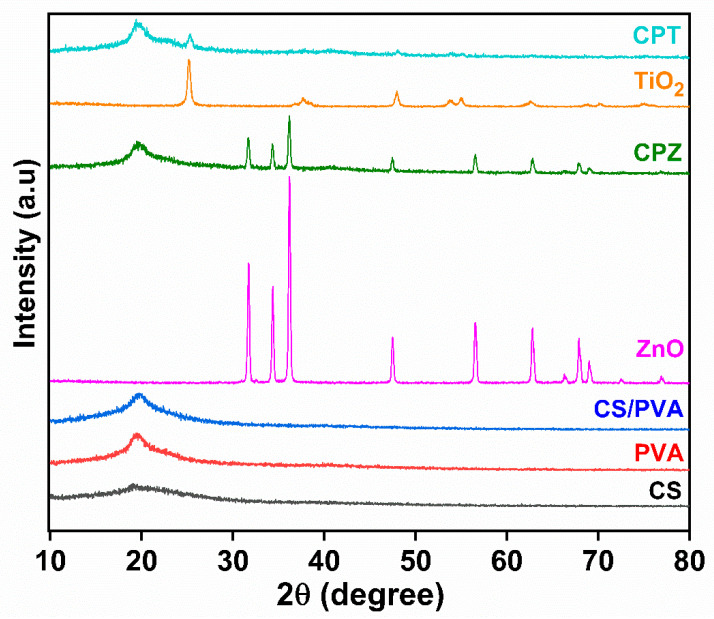
X-ray diffraction pattern of CS-based CPZ and CPT BNCs.

The XRD pattern of both the BNCs was analyzed as depicted in Figure 5. Pure CS exhibited peaks at 2θ = 11.3° and 20°, and pure PVA at 2θ = 19.7°, while the blending of the CS/PVA films exhibited peaks at 2θ = 13.24° and 19.4°. Aside from that, pure ZnO NPs showed peaks with 2θ at 31.68°, 34.35°, 36.18°, 47.5°, 56.54°, 62.78°, 67.88°, and 68.99°, which attributed to the lattice plane (100), (002), (101), (102), (110), (103), (200), and (112), respectively, with wurtzite crystal having hexagonal lattice matches with JCPDS no. 36-1451. The diffraction pattern of CPZ BNCs shows a characteristic peak of both blended CS/PVA as well as ZnO NPs at 2θ = 19.4°, 28.44°, 31.68°, 34.35°, 36.18°, 47.5°, 56.54°, 62.78°, 67.88°, and 68.99°, revealing a successful incorporation of the latter inside the polymer matrix with decreased intensity. Similarly, in the case of TiO_2_, pure TiO_2_ NPs exhibited major diffraction peaks at 2θ = 25.2° and minor low-intensity peaks at 2θ = 37.69°, 47.97°, 53.81°, 55.01°, 62.6°, 68.79°, 70.19°, and 75.01°, which attributed to the lattice plane (101), (004), (200), (105), (211), (204), (116), (220), and (107), respectively, with the anatase crystal structure having a tetragonal lattice match with JCPDS no. 21-1272. The CPT BNCs revealed major peaks at 2θ = 19.4° and 25.2°, along with other low-intensity peaks at 2θ = 37.69°, 47.97°, 53.81°, 55.01°, 62.6°, 68.79°, 70.19°, and 75.01° as compared to their pure material, which again indicated the immobilization of TiO_2_ NPs in the blended films. The average size of both the NPs has been calculated by using the Debye–Scherrer Equation as follows:

(4) $$D=\frac{0.9\;\lambda }{\beta \;cos\theta }$$

where ‘*D*’ is the average particle size or crystallite size, ‘*λ*’ is the wavelength of X-ray, ‘*β*’ is full width at half maximum (FWHM), and *θ* is the diffraction angle. It was found that the average crystallite size of ZnO NPs was 28.76 nm, while that of TiO_2_ NPs was 21.26 nm.

### 4.8. HR-FESEM Analysis

**Figure 6:**
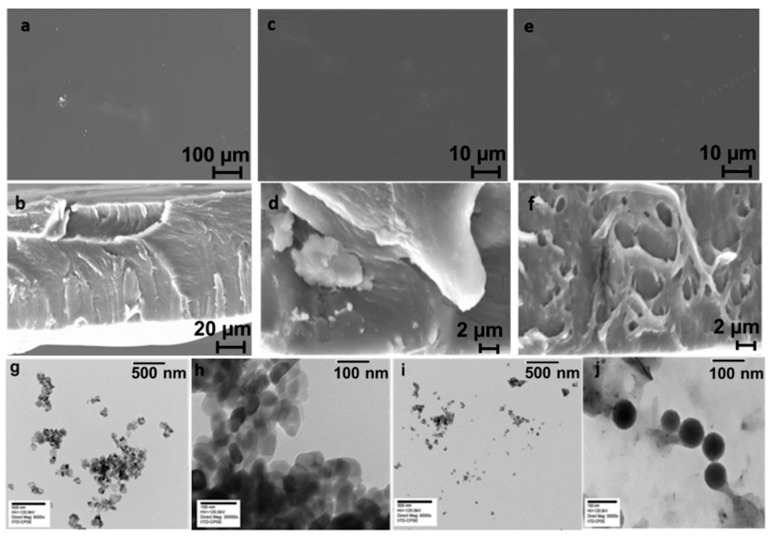
SEM images of surface and transverse section of (**a**,**b**) CS/PVA, (**c**,**d**) CPZ, and (**e**,**f**) CPT and TEM micrographs of distribution of (**g**,**h**) ZnO NPs and (**i**,**j**) TiO_2_ NPs.

The surface and longitudinal cross-sectioned morphology of the metal oxide BNC films are shown in Figure 6a–f. As depicted in Figure 6a,b, the blended CS/PVA polymeric film was uniform, smooth, continuous, and homogeneous with linear structural integrity and without any interface layer. This confirms the uniform blending and distribution of CS and PVA throughout the film, indicating their better compatibility. This was mainly due to the H-bonding between the –OH and –NH_2_ groups of CS with the –OH group of PVA. Hajji et al. observed a similar compatibility of the blended CS/PVA polymeric films [19]. On the other hand, in the case of the CPZ BNC film, the aggregation of ZnO NPs in the blended polymeric film was observed, whereas the smooth and homogeneous dispersion of TiO_2_ NPs was observed in the case of the CPT BNCs polymeric matrix, as shown in Figure 6e,f. This indicates the phase compatibility of the blended components and the successful incorporation of ZnO and TiO_2_ NPs inside the polymeric CS/PVA films. Additionally, the aggregation of immobilized ZnO NPs was recently reported in the chitosan-based polymeric films as well as the dispersed TiO_2_ NPs incorporation within the incompatible whey protein-based films [37].

### 4.9. TEM Analysis

Figure 6g–j illustrates the TEM images of ZnO and TiO_2_ NPs, respectively. Aside from that, Figure 6g,h shows that ZnO NPs are present in an aggregated form. Although some of the ZnO NPs overlapped with each other and were found to have an overall dispersion effect in both the concentration of ZnO NPs. The shape of ZnO NPs was found to be hexagonal and spherical at some places with an average particle size in the range of 20–30 nm, which is in good agreement with the XRD results. The higher agglomeration of small-sized NPs can be seen in the HR-FESEM images. These ZnO NPs are agglomerated and immobilized on the surface of the polymeric matrix consisting of CS and PVA, but they also contain a small number of dispersed NPs, making them appropriate for further biological activities. Similarly, the results for the use of ZnO NPs were reported in the literature [38]. Also, it has been evident from the images that the spherically shaped TiO_2_ NPs are found to be well dispersed in the CS/PVA polymeric matrix (Figure 6i,j). Owing to the high electron density of TiO_2_ NPs, the immobilization and distribution of crystalline TiO_2_ NPs on the surface of blended polymeric CS/PVA are illustrated with bright areas corresponding to the amorphous CS while the dark areas represent the crystalline TiO_2_ NPs. The TEM images revealed the average particle size of around 20 nm of the TiO_2_ NPs, which is found worthy of the XRD results. There are available reports which revealed similar results for the TiO_2_ NPs [39].

### 4.10. Antimicrobial Studies

The results from Table 3 display consistent antibacterial activity against the bacterial pathogen *Escherichia coli* under consideration. It is evident from Table 3 that the polymeric compounds exhibited a lesser zone of inhibition, and therefore, a low antibacterial efficacy as compared to the BNCs. Out of the metal oxide BNCs, CPZ was found to have a large zone of inhibition (5.3 ± 0.1 mm) with increased concentration, and thus exhibited higher antibacterial activity than the CPT BNCs, which shows less antibacterial efficacy. This is mainly observed due to the rupture of the bacterial cell wall which leads to the dysfunction of DNA and hence caused the death of the bacterial cell because of the participation of metal oxide [40]. However, at a low concentration of 50 µg/mL, CPT BNCs exhibited a greater zone of inhibition (2.3 ± 0.03 mm) than CPZ BNCs. Boura-Theodoridou demonstrated significant inhibition of the growth of microorganisms due to Zn^2+^ dissolution in the Chitosan/ZnO films [41]. The overall high antibacterial efficacy of metal oxide-immobilized polymeric films strongly recommends their application in the biomedical field of sciences.

### 4.11. Antioxidant Studies

**Figure 7:**
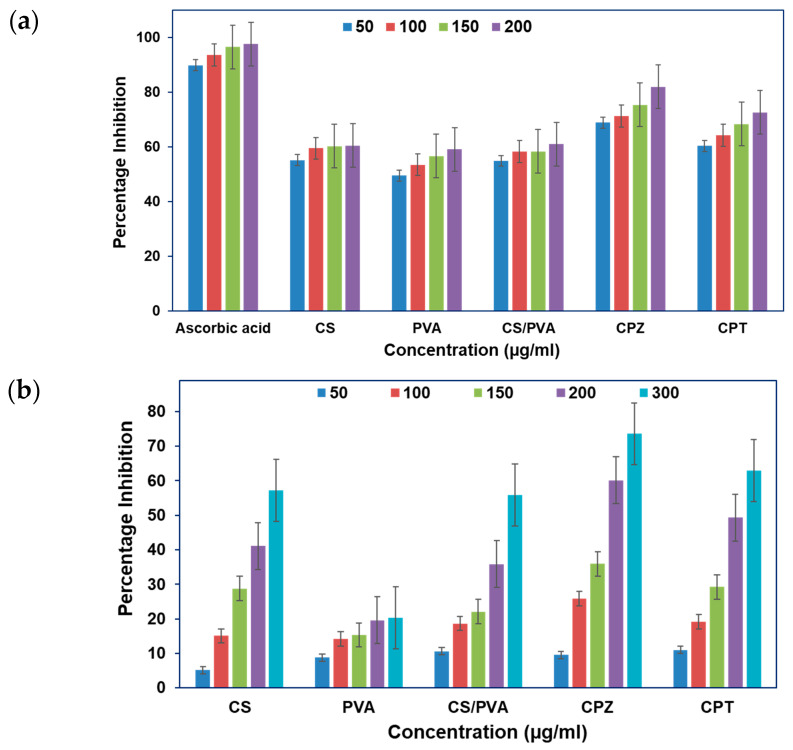
(**a**) Antioxidant activity of CPZ and CPT BNCs and (**b**) anticancer activity of ZnO and TiO_2_ BNCs against the A431 skin cancer cell line. Results are shown as the mean ± SD (*n* = 3), different letters in the superscript show significant differences at *p* < 0.05.

The DPPH radical scavenging potential of polymeric films and BNCs is observed in Figure 7a. Pure CS shows the highest scavenging activity (~85%) among all the polymeric BNCs. Aside from that, CPZ and CPT BNCs possess scavenging activity of approximately 75% and 70%, respectively, in a dose-dependent manner and were higher than the blended polymeric CS/PVA films (~50%). Similarly, Safawo et al. reported that the increased concentration of NPs can increase their scavenging ability [42]. The ascorbic acid was taken as a reference compound. The results indicate that the NPs’ immobilized BNCs exhibit high free radical scavenging ability. Niska et al. reported the scavenging effect of TiO_2_ NPs on the family of SOD enzymes and noticed the oxidatively induced cell death [43].

### 4.12. Cell Viability or Anticancer Studies

The cell viability or anticancer activities reported for both the fabricated metal oxide BNCs were subjected to the skin cancer cell line A431 under varying concentrations (50, 100, 150, 200, and 300 µg/mL). As evident from Figure 7b, all the BNCs showed a dose-dependent response in terms of cell viability. Meanwhile, it has been found that pure CS exhibited the highest percentage cell inhibition (75%), contrary to pure PVA. However, the blended CS/PVA exhibited a moderate response of around 50%. Furthermore, the CPZ BNCs possess a higher percentage cell inhibition (70%) as compared to CPT BNCs (61%). This may be due to the structural difference which leads to more interaction of the ZnO NPs with the cancerous cells than the TiO_2_ NPs, and therefore, the CPZ BNCs put forth high anticancer activity as compared to CPT BNCs. The metal oxide NPs, such as TiO_2_ and ZnO, profoundly expressed the effective dose-dependent tumor inhibition against different cancer cell lines, such as breast cancer, MCF-7, and liver cancer (HepG2), but the comparative study for skin cancer has not been reported yet [44]. Table 4 shows recent studies carried out with chitosan-based BNCs for various biomedical applications. This comparative analysis demonstrates that the BNCs synthesized in the current study provide an improved option in comparison to the alternatives.

In general, the basic mechanism for the antioxidant and anticancer activities of the BNCs is apoptosis. Apoptosis is blocked by normal ATP levels; hence ATP is assumed to govern apoptotic signals [45]. As a result, a decrease in ATP levels following the addition of ZnO BNCs (CPZ) might be blamed for triggering apoptosis via caspase pathways and resulting in cell death. The TiO_2_ BNCs (CPT) were likewise shown to be dose-dependent and responsible for the emission of ROS. ROS are typically harmful to cancer cells and are thought to be essential in the cellular death process [46]. Furthermore, when Zn BNCs interacted with the cancer cells, the most typical damage were cell shrinkage and death, compromising cell membrane integrity, disturbing ATP generation, and obstructing electron transport [45].

**Table 4:** Recent studies carried out using chitosan-based BNCs for various biomedical applications.

Biopolymer	Modifier	Application	Biological Activity				Reference
Chitosan	Ag	Antimicrobial, antioxidant, and anticervical cancer activity	Cervical cancer HeLa cell line viability = 22%				[11]
	Polylactic acid	Enzymatic hydrolysis	Cassava waste hydrolysis with a conversion rate = 0.99				[47]
	ZnO	Antibiofouling and water disinfection	Antibacterial = 85.6% Antifungal = 92% (*A. fumigatus*); 77.7% (*F. solani*)				[10]
	PVA	Food packaging	Antioxidant activity = 41.1 ± 1.17				[16]
	Silk fiber and PVA	Tissue engineering	Haemolysis inhibition = >80%				[17]
	PVA and ZnO	Dye removal	AB 1 dye removal = 86% Cell viability = >120% (CS/PVA/ZnO-10)				[20]
	ZnO and TiO_2_	Antimicrobial, antioxidant, and anticancer activity	BNCs	Antibacterial activity Zone of inhibition (in mm)	Scavenging activity	Skin cancer cell line A431 inhibition (%)	**Present study**
			CPZ	5.3 ± 0.1	75	70	
			CPT	2.3 ± 0.03	70	61	

## 5. Conclusions

The BNCs possessing CS-PVA impregnated with ZnO and TiO_2_ NPs were successfully synthesized. The concentration of ZnO and TiO_2_ NPs was optimized and the effect of both the NPs on the overall properties of the obtained BNCs was clarified. The addition of NPs (ZnO and TiO_2_) was found to be effective in increasing antimicrobial, antioxidant, and anticancer activities. The CPZ BNCs possessed high scavenging activity of approximately 75%, while CPT BNC possessed 70% in a dose-dependent manner. Similarly, the CPZ BNCs (70%) illustrated higher percent inhibition of the skin cancer cell line, A431, in a dose-dependent manner than the CPT BNCs (61%). Therefore, this method for the synthesis of CS/PVA-based BNCs films showed effective anticancer, antimicrobial, and antioxidant activities. Thus, BNCs are potential candidates for applications in cancer therapy. The findings of this study provide useful information for further research to explore the fields of biomedical and biological sciences.

## Figures and Tables

**Table 1:** Composition of BNCs.

BNCs	CS (wt.%)	PVA (wt.%)	ZnO (wt.%)	TiO_2_ (wt.%)
**CPZ1**	1	5	0.1	-
**CPZ2**			0.5	-
**CPT1**			-	0.1
**CPT2**			-	0.5

**Table 2:** Physical appearance, thickness, and % moisture content of the polymeric films.

Sample	Moisture Content	Thickness (mm)	Physical Appearance
**PVA**	42.4 ± 1.73%	0.16 ± 0.01 ^a^	Colorless, Transparent
**CS**	20 ± 2.25%	0.15 ± 0.02 ^b^	Yellowish
**CS/PVA**	15.8 ± 2.2%	0.13 ± 0.02 ^b^	Yellowish
**CPZ1**	13.9 ± 0.23%	0.12 ± 0.02 ^b^	Light Yellowish
**CPZ2**	14.8 ± 0.36%	0.11 ± 0.01 ^a^	Light Yellowish
**CPT1**	10.6 ± 0.50%	0.09 ± 0.01 ^a^	Light Yellowish
**CPT2**	9.4 ± 0.32%	0.08 ± 0.0 ^c^	Light Yellowish

Results are shown as the mean *±* SD (*n* = 3), different letters in the superscript show significant differences at *p* < 0.05.

**Table 3:** Zone of inhibition observed by CS, CS/PVA, CPZ, and CPT BNCs against *E. coli*.

	Conc. µg/mL	50	100	150	200	300
Samples						
**CS**		1.6 ± 0.05 ^a^	2 ± 0.03 ^b^	1.6 ± 0.05 ^a^	2.6 ± 0.05 ^a^	3 ± 0.05 ^a^
**CS/PVA**		2.3 ± 0.03 ^b^	2.3 ± 0.03 ^b^	2.6 ± 0.03 ^b^	3.6 ± 0.05 ^a^	4 ± 0.05 ^a^
**CPZ BNCs**		1.3 ± 0.1 ^e^	2.6 ± 0.15 ^e^	3.3 ± 0.05 ^a^	5.0 ± 0.06 ^d^	5.3 ± 0.1 ^e^
**CPT BNCs**		2.3 ± 0.03 ^b^	3.3 ± 0.06 ^d^	3.3 ± 0.03 ^b^	3.3 ± 0.08 ^c^	4.6 ± 0.08 ^c^

Data are the means of the three replicates (*n* = 3) ± standard error, different letters in the superscript show significant differences at *p* < 0.05.

## Data Availability

The data that support the findings of this study are available on request from the corresponding author.

## Supplementary Materials

The following supporting information can be downloaded at: https://www.mdpi.com/article/10.3390/polym15163477/s1.

## Abbreviations

CS	Chitosan
PVA	Polyvinyl Alcohol
BNCs	Bionanocomposites
NP	Nanoparticles
CPZ	CS/PVA/ZnO
CPT	CS/PVA/TiO_2_
