# Supplementary material for: Comparative Study of ZnO-and-TiO2-Nanoparticles-Functionalized Polyvinyl Alcohol/Chitosan Bionanocomposites for Multifunctional Biomedical Applications
Source: Polymers (Basel). 2023 Aug 19;15(16):3477. doi: 10.3390/polym15163477 (PMC10459413; doi:10.3390/polym15163477)
Supplement: Supplementary file 1 [file polymers-15-03477-s001.zip › polymers-2506777-SI.pdf]

polymers

Research Article

Comparative Study of ZnO-and-TiO_2-Nanoparticles-Functionalized Polyvinyl Alcohol/Chitosan Bionanocomposites for Multifunctional Biomedical Applications

Annu [1,2,*], Zafar Iqbal Bhat [3], Khalid Imtiyaz [3], M. Moshahid A. Rizvi [3], Saiqa Ikram [1,*] and Dong Kil Shin [2,*]

[1] Thin-Film Engineering and Materials Laboratory, School of Mechanical Engineering, Yeungnam University, Gyeongsan 38541, Republic of Korea
[2] Bio/Polymers Research Laboratory, Department of Chemistry, Jamia Millia Islamia, New Delhi 110025, India
[3] Department of Biosciences, Jamia Millia Islamia, New Delhi 110025, India
* Correspondence: annuchem92@gmail.com or drannu@yu.ac.kr (A.); sikram@jmi.ac.in (S.I.); dkshin@yu.ac.kr (D.K.S.)

Supplementary Figures:

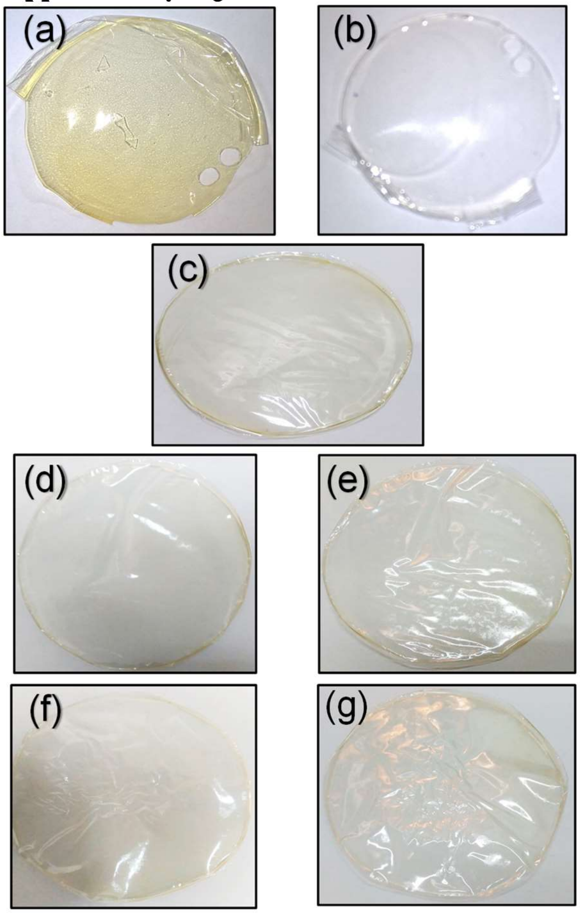

Figure S1: Visual representation of (a) pure CS, (b) pure PVA, (c) CS/PVA, (d) CPZ1, (e) CPZ2, (f) CPT1, and (g) CPT2

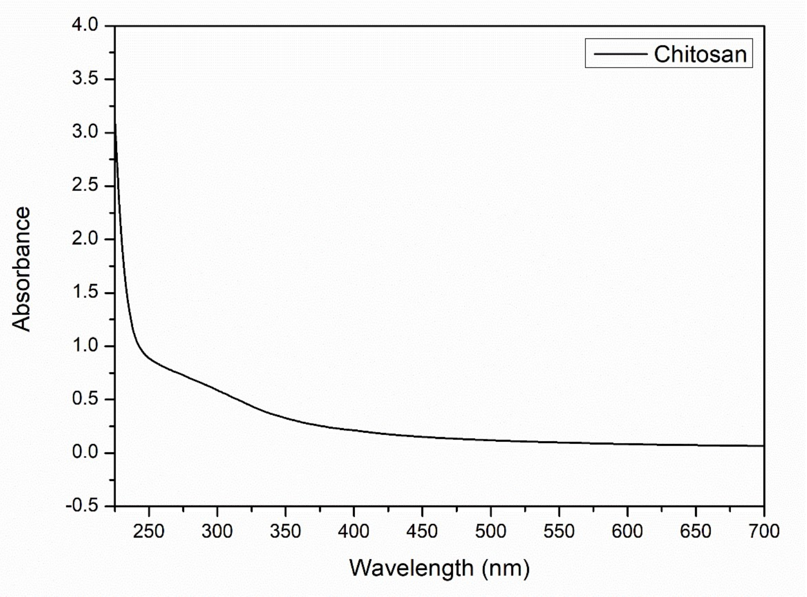

Figure S2: UV-Vis spectra of pure CS
